# Ringworm Fungus in a Strange Situation

**Published:** 1908-07-11

**Authors:** Charles Dibren

**Affiliations:** Maldon, Essex.


### RINGWORM FUNGUS IN A STRANGE SITUATION.

Sir,—Would you grant me space in your journal? About three years ago I mounted a specimen of a mouse's testicle, and there discovered a most perfect specimen of pure culture tricophyton. This I have compared with some pure cultures sent me by a doctor in London. The specimens from the mouse and from the cultivation are in every detail alike. I should like to hear the opinion of other men on this subject. I cannot but think it is something remarkable to find tricophyton in its pure state in this section of the mouse. I should be pleased to submit the two specimens to any body of gentlemen. Believe me, yours truly,

Maldon, Essex. Charles Dibben.

